# A Polyvalent Broad-Spectrum *Escherichia* Phage *Tequatrovirus* EP01 Capable of Controlling *Salmonella* and *Escherichia coli* Contamination in Foods

**DOI:** 10.3390/v14020286

**Published:** 2022-01-29

**Authors:** Yuqing Zhou, Lei Li, Kaiou Han, Leping Wang, Yajie Cao, Dongxin Ma, Xiaoye Wang

**Affiliations:** College of Animal Science and Technology, Guangxi University, Nanning 530004, China; 1918302044@st.gxu.edu.cn (Y.Z.); 2018302015@st.gxu.edu.cn (L.L.); 201839301@st.gxu.edu.cn (K.H.); 2018393051@st.gxu.edu.cn (L.W.); 2018393003@st.gxu.edu.cn (Y.C.); 1918302018@st.gxu.edu.cn (D.M.)

**Keywords:** *Salmonella*, *Escherichia coli*, food contamination, polyvalent broad-spectrum phage

## Abstract

*Salmonella* and *Escherichia coli* (*E. coli*) food contamination could lead to serious foodborne diseases. The gradual increase in the incidence of foodborne disease invokes new and efficient methods to limit food pathogenic microorganism contamination. In this study, a polyvalent broad-spectrum *Escherichia* phage named *Tequatrovirus* EP01 was isolated from pig farm sewage. It could lyse both *Salmonella Enteritidis* (*S. Enteritidis*) and *E. coli* and exhibited broad host range. EP01 possessed a short latent period (10 min), a large burst size (80 PFU/cell), and moderate pH stability (4–10) and appropriate thermal tolerance (30–80 °C). Electron microscopy and genome sequence revealed that EP01 belonged to T4-like viruses genus, *Myoviridae* family. EP01 harbored 12 CDSs associated with receptor-binding proteins and lacked virulence genes and drug resistance genes. We tested the inhibitory effect of EP01 on *S. Enteritidis*, *E. coli* O157:H7, *E. coli* O114:K90 (B90), and *E. coli* O142:K86 (B) in liquid broth medium (LB). EP01 could significantly reduce the counts of all tested strains compared with phage-free groups. We further examined the effectiveness of EP01 in controlling bacterial contamination in two kinds of foods (meat and milk) contaminated with *S. Enteritidis*, *E. coli* O157:H7, *E. coli* O114:K90 (B90), and *E. coli* O142:K86 (B), respectively. EP01 significantly reduced the viable counts of all the tested bacteria (2.18–6.55 log_10_ CFU/sample, *p* < 0.05). A significant reduction of 6.55 log_10_ CFU/cm^2^ (*p* < 0.001) in bacterial counts on the surface of meat was observed with EP01 treatment. Addition of EP01 at MOI of 1 decreased the counts of bacteria by 4.3 log_10_ CFU/mL (*p* < 0.001) in milk. Generally, the inhibitory effect exhibited more stable at 4 °C than that at 28 °C, whereas the opposite results were observed in milk. The antibacterial effects were better at MOI of 1 than that at MOI of 0.001. These results suggests that phage EP01-based method is a promising strategy of controlling *Salmonella* and *Escherichia coli* pathogens to limit microbial food contamination.

## 1. Introduction

Bacterial food contamination is the main cause of foodborne diseases. *Salmonella* and *Escherichia coli* (*E. coli*) are common bacteria that result in food contamination. *Salmonella* infections can cause several symptoms including headache, fever, diarrhea, and abdominal pain [1]. Some specific serotypes of *E. coli* (O157:H7, O114:K90 (B90), O142:K86 (B)) are pathogenic and result in diarrhea, edema, haemorrhagic colitis, urinary infection, and septicemia. At present, over 2500 serovars of *Salmonella* have been identified. *Salmonella Enteritidis* (*S. Enteritidis*) is one of the most dangerous serovars since it was reported to be the major cause of over 50% of salmonellosis [2]. After the first report in the early 1980s, *E. coli* O157:H7 that caused foodborne diseases outbreaks has attracted more and more attention from academics and experts worldwide [3]. *S. Enteritidis* and *E. coli* were commonly found in contaminated water, dairy products, pork, vegetables, and fruits and resulted in foodborne diseases frequently [4].

Physical, chemical, and biological methods can be used to control bacterial contamination in foods. However, various weakness and adverse impacts pose challenges for their application. In the germicidal process, physical methods like irradiation might cause nutritive loss and produce unpleasant odor [5]. Chemical disinfectant such as chlorine is not environmentally friendly and harmful to public health. Chlorine can even form carcinogenic compounds under certain conditions [6]. Furthermore, heavy use of chlorine can induce the development of drug-resistant bacteria [7]. Besides, overuse of antibiotics has led to increased drug resistance of bacteria including *Salmonella* and *E. coli* over the past few decades [8]. We urgently need to develop new and effective methods to control *Salmonella* and *E. coli* contamination in foods to reduce the incidence of foodborne diseases.

Phage-based biocontrol methods are increasingly considered as prospective strategies for reducing bacterial food contamination. Phages have advantages of easy availability, specificity, harmlessness to the public, and being environmentally friendly compared to conventional antimicrobials. Many experimental studies have reported applying phages to control *Salmonella* and *E. coli* contamination in various foods. A specific *Salmonella* phage D1-2 could effectively reduce the viable counts of MDR *S. Enteritidis* 11561 and *S. Typhimurium* SJTUF 13277 in liquid eggs white and egg yolk at different temperatures [9]. Phages are used to inactive *E. coli* O157:H7 attached to spinach harvester blade with high efficiency [10]. A broad-spectrum *Salmonella* phage LPST10 could cause viable counts reductions (0.92–5.12 log_10_ CFU/sample) in *S. Typhimurium* and *S. Enteritidis* in sausages and lettuce [11]. Besides, phages could effectively control the contamination of other foodborne pathogens. For example, a phage OMN had successfully controlled *Vibrio parahaemolyticus* in oysters [12]. However, most phages could only inhibit the growth of bacteria within one genus, which limits the wide use of phages in food industry. As a result, the concepts of phage cocktails (mixture of multiple phages) and polyvalent phages (a single phage targets multiple bacterial species) are proposed. Previous studies have demonstrated the effectiveness of phage cocktails and polyvalent phages inhibiting the growth of *Salmonella* and *E. coli* in different food matrices. *E. coli* O157:H7 and *Salmonella* are well controlled using bacteriophage cocktail on contaminated fresh fruits and vegetables [13]. Polyvalent phage PS5 exhibited the great efficacy inhibiting the growth of *S. Enteritidis*, *S. Typhimurium*, and *E. coli* O157:H7 in chicken skin, raw beef, fresh lettuce, pasteurized milk, and liquid egg [14].

Our study isolated and identified a polyvalent broad-spectrum phage named EP01. We determined its host range, biological characteristics, and the lytic capacity against hosts in liquid broth medium (LB). The major objective of this study is to assess the effectiveness of a polyvalent broad-spectrum phage to biocontrol *Salmonella* and *E. coli* contamination in two different foods.

## 2. Materials and Methods

### 2.1. Bacterial Strains, Phages and Growth Conditions

Seventy-six bacterial strains were used in this study (Supplementary Table S1). *E. coli* O8: K88 CVCC1527 (No.1511C0006000001663), *E. coli* O157: H7 CVCC4050 (No.1511C0006000003806), *Salmonella enteritidis* CVCC1806 (No.1511C0006000000119), and *Salmonella typhimurium* CVCC3384 (No.1511C0006D00000043) were purchased from China Veterinary Culture Collection Center (Beijing, China). The other strains were isolated from livestock and poultry farms (Supplementary Table S1). All the bacterial isolates were cultured in LB broth at 37 °C for 8–12 h and kept at −80 °C in LB provided with 20% *v*/*v* glycerol.

Six phages named as *Escherichia* phage *Tequatrovirus* EP01, *Escherichia* phage *Mosigvirus* EP02, *Escherichia* phage *Mosigvirus* EP03, *Escherichia* phage *Mosigvirus* EP04, *Escherichia* phage *Mosigvirus* EP05, and *Escherichia* phage *Felixounavirus* EP06 were isolated from pig farm sewage in Nanning, Guangxi, China. These phages were purified and amplified using *Salmonella*, *E. coli* O157:H7, and *E. coli* O8:K88 as host strains. Purified phages were stored in 20% glycerol at −80 °C. All the bacterial strains and phages were preserved in our laboratory.

### 2.2. Host Range of Isolated Phages

The host range of isolated phages was determined against 59 *Escherichia coli* strains, 4 *Salmonella* strains, 10 *Klebsiella pneumoniae* strains, 1 *Pseudomonas aeruginosa* strain, and 2 *Proteus mirabilis* strains by the spot test [15]. Suspensions of the tested strains (100 µL) were mixed with LB containing 0.6% agar (3 mL) serving as the overlay and LB containing 1.2% agar (15 mL) was served as the bottom layer. Then 5 µL of phage lysates (10^9^ PFU/mL) were spotted onto a double-layer agar plate containing the lawns of target strains and incubated at 37 °C for 18–24 h.

### 2.3. Morphological Analysis

Phage suspension (10^10^ PFU/mL) was ultracentrifuged (40,000 rpm, 4 °C) for 1 h and resuspended in 0.1 mol/L ammonium acetate. The copper grid for transmission electron microscopy (TEM) was immersed in EP01 suspension for 10 min, then stained with phosphotungstic acid solution (volume fraction of 2%, pH = 7) for 10 min. The morphology of EP01 was observed using TEM (Hitachi High-Tech Co., Ltd., Tokyo, Japan).

### 2.4. Optimal Multiplicity of Infection Test

Multiplicity of infection (MOI) refers to the ratio of phages added to host bacteria or MOI input [16]. According to a certain MOI (0.0001, 0.001, 0.01, 0.1, 1, 10, 100), 1 mL of phage suspension was mixed with 1 mL of host bacteria GXEC-N01 in LB, the mixture was then incubated at 37 °C (180 rpm) for 4 h. After centrifugation (12,000 rpm, 4 °C) for 10 min, double-layer agar plates methods were used to determine the phage titers [17]. The multiplicity of infection with the highest titer is the optimal MOI of the phage.

### 2.5. One-Step Growth Test

Latent period and burst size were examined by one-step growth experiments. One-step growth tests were performed by a modified method described elsewhere [18]. EP01 was mixed with host bacteria GXEC-N01 at the optimal MOI of 0.001. The mixture was incubated at 37 °C for 15 min and then centrifuged (12,000 rpm, 1 min). Then, phage pellet was washed twice with LB broth and resuspended in 10 mL of pre-warmed LB. The suspension was immediately incubated at 37 °C for 3 h. Every 10 min (up to 120 min), samples were taken and centrifuged (12,000 rpm, 4 °C) for 2 min. The phage titers were determined by methods of double-layer agar plate to obtain one-step curve.

### 2.6. Thermal and pH Stability

To assess the thermal stability, phage lysates were inoculated at 30 °C, 40 °C, 50 °C, 60 °C, 70 °C, and 80 °C. Samples were taken at 30-min intervals (up to 1 h) and phage titers were then determined by double-layered agar plate method [19]. For pH stability, phage lysates were added to saline magnesium (SM) buffer at different pH (2–12) and then incubated at 37 °C for 2 h. The phage titers were determined as mentioned above [17].

### 2.7. Extraction and Analysis of Phage Tequatrovirus EP01 Genome

Genomic DNA of EP01 was extracted from high-titer phage suspension (10^10^ PFU/mL) using previously validated method [17]. The construction of sequencing library and the control of sequencing data quality were performed by Personalbio (Nanjing Personal Biotechnology Co., Ltd., Nanjing, China). The coding DNA sequences (CDSs) were predicted and annotated using the RAST server 2.0 [20]. Putative virulence factors and antimicrobial resistance genes were screened by the Virulence Factor Database [21] and Comprehensive Antibiotic Resistance Database [22], respectively. Comparative circular genome map of EP01 genome was depicted by CGView Server V1 [23]. Phylogenetic analysis was constructed based on the major capsid proteins of 28 phages including EP01. Sequence alignment was performed by ClustalW 2.1 [24] and tree visualization was performed with FigTree 1.4.4 (http://tree.bio.ed.ac.uk/software/figtree/, accessed on 12 January 2021). The phylogenetic tree was generated in Mega X 10.2.5 using neighbor joining method with *p* distance values and bootstrap replicate of 500 [25].

### 2.8. Bacterial Challenge Assay against S. Enteritidis, E. coli O157:H7, E. coli O114:K90 (B90), and E. coli O142:K86 (B) Contamination in LB Broth

Three milliliter of *S. Enteritidis* GXSM-N02 suspension (10^8^ CFU/mL) was mixed with 5 mL LB and 3 mL EP01 suspension. Each *E. coli* strain (*E. coli* O157:H7, *E. coli* O114:K90 (B90), and *E. coli* O142:K86 (B)) was added to phage lysates at MOI of 0.001 and 1, respectively. Three milliliter of host suspension (10^5^ PFU/mL) mixed with 8 mL of LB was set as a positive control. The lytic capacity of the phage against each strain was determined by measuring the growth of the host (OD_600 nm_) at a one-hour interval up to 5 h at 37 °C.

### 2.9. Bacterial Challenge Assay against S. Enteritidis, E. coli O157:H7, E. coli O114:K90 (B90), and E. coli O142:K86 (B) Contamination on the Surface of Raw Meat and in Fresh Milk

#### 2.9.1. Food Samples Preparation

Fresh skim milk and raw meat were purchased from local market and stored at 4 °C. The raw meat was cut into small pieces (3 × 3 cm^2^) using a sterile knife, placed on a sterile plate, and then subjected to UV light treatment for 30 min to eliminate bacteria on the raw meat surface.

#### 2.9.2. Application of Phage *Tequatrovirus* EP01 on the Surface of Raw Meat and in Fresh Milk

The surface of meat slice (3 × 3 cm^2^) was artificially contaminated with 25 µL of host suspension (*S. Enteritidis*, *E. coli* O157:H7, *E. coli* O114:K90 (B90) or *E. coli* O142:K86 (B), 2 × 10^8^ PFU/mL). The contaminated samples were allowed to dry in a biological safety cabinet for 30 min before phage treatment. For phage treatment, the surface of contaminated samples was spotted with 50 µL of phage suspension at MOIs of 0.001 and 1 and then incubated at 4 °C and 28 °C, respectively. In phage free control groups, Tris-SM buffer (50 mM Tris-HCl, 8 mM magnesium sulfate, 100 mM sodium chloride, and 0.01% gelatin, pH 7.5) was used instead of phage lysate. After 2, 4, 6, and 24 h of incubation, the samples were withdrawn and homogenized with Phosphate Buffer Saline buffer (PBS). Then, 1 mL of the homogenate was taken out and serially diluted using PBS buffer. The dilution (100 µL) was plated on Eosin-Methylene Blue (EMB) agar to determine the bacterial counts using the bacterial plate counting methods.

The fresh milk was artificially contaminated with host suspension (100 µL, 10^8^ CFU/mL). For phage treatment, EP01 (100 µL) at MOIs of 0.001 and 1 was then added to the contaminated milk and kept at 4 °C and 28 °C, respectively. In positive control groups, Tris-SM buffer was used instead of phage lysate. After incubation for 2, 4, 6, 24 h, 500 µL of the mixture of each group was withdrawn and serially diluted using PBS buffer. Then the bacterial counts were measured by plating method as described above.

Because phage titers corresponding to *S. Enteritidis* were unable to be determined, the experiments of antibacterial effect in foods against *S. Enteritidis* could only set up two phage treatment groups including two different temperatures (4 °C and 28 °C).

### 2.10. Statistical Analysis

The experimental design was randomized entirely and all the experiments were conducted in triplicates. Statistical analysis was performed using SPSS statistical software. One way ANOVA was used to determine the significance among groups at a significance level (*p* < 0.05). The interval of confidence was set at 95%.

## 3. Results

### 3.1. Phage Tequatrovirus EP01 Exhibited Polyvalent Broad Spectrum

**Figure 1:**
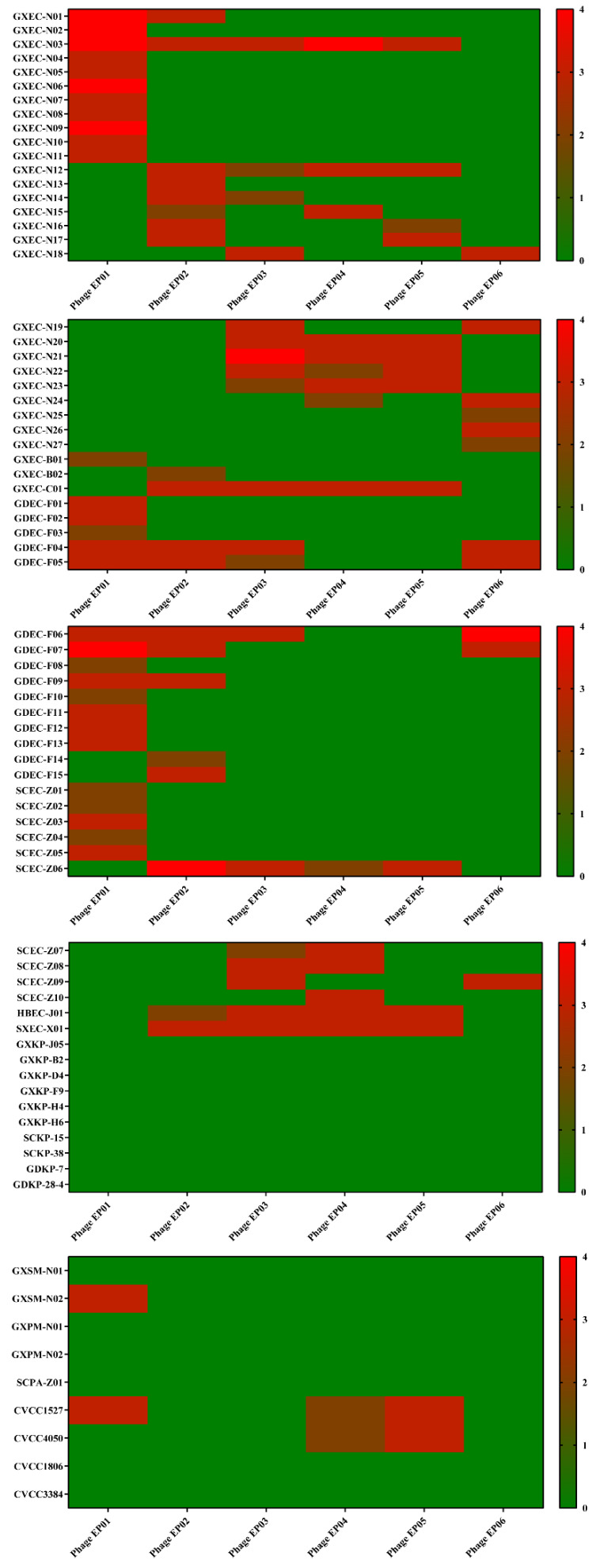
Host spectrum of 6 isolated broad-spectrum bacteriophages to 60 *Escherichia coli* isolates, 4 preserved *Salmonella* isolates, 10 *Klebsiella pneumoniae* isolates, 2 preserved *Proteus mirabilis* isolates, and 1 *Pseudomonas aeruginosa* isolates. Lytic capability is indicated by heat maps. Numbers from 0 to 4 correspond to colors from green to red; “+4” indicates a completely clear plaque; “+3” indicates a generally clear plaque with the faint hazy background; “+2” indicates obvious turbidity throughout clear lytic zone; “+1” indicates an individually opaque plaque; “0” indicates no lytic zone.

Six phages were isolated from sewage and named as phage *Tequatrovirus* EP01, *Mosigvirus* EP02, *Mosigvirus* EP03, *Mosigvirus* EP04, *Mosigvirus* EP05, and *Felixounavirus* EP06 (designated as EP01, EP02, EP03, EP04, EP05, and EP06), respectively. The morphology of these phages on double-layer agar plates were clean and bright with neat edges, exhibiting typical characteristics of lytic phages (Supplementary Figure S1). The host spectrum of these phages was tested against 76 strains (Supplementary Table S1). Among these phages, EP01 could infect 31 of the 59 tested *E. coli* strains and 1 of 4 tested *Salmonella* strains (Figure 1). EP01 showed a weak lytic capability to strains from other species like *Salmonella*, *Klebsiella pneumoniae*, *Pseudomonas aeruginosa*, and *Proteus mirabilis* (Figure 1). Plaque phenotype of EP01 varies on different host strains (Supplementary Figure S2). On *E. coli* host strains, the plaques were clear and translucent with different diameter (Supplementary Figure S2). EP01 are able to form bright spots using *Salmonella* as host strain but could not form plaque on double-layer agar plates (Supplementary Figure S2).

### 3.2. Biological Characteristics Analysis

**Figure 2:**
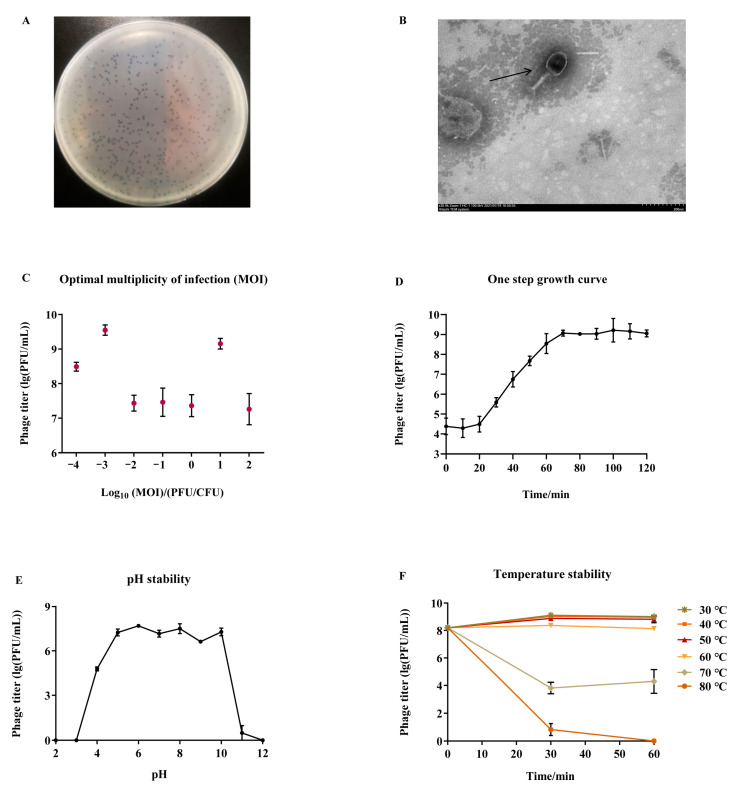
Biological characterization of phage *Tequatrovirus* EP01. (**A**) Morphology on double−layer agar plates. (**B**) Transmission electron micrograph (30,000×). (**C**) Optimal multiplicity of infection. (**D**) One−step growth curve. (**E**) Stability at different pH from 2 to 12. (**F**) Stability at different temperatures from 30~80 °C.

The plaque morphology observed on plates showed that EP01 was able to form a clean and bright plaque (Figure 2A). Furthermore, the plaque was 3 mm in diameter (Figure 2A). TEM showed that EP01 had an icosahedral shape with a stereo-symmetric head and a long, contractile tail (Figure 2B). The head is a typical regular polyhedral shape with a diameter of 45.37 nm (Figure 2B). The tail length and diameter are approximately 150 nm and 10 nm, respectively (Figure 2B). According to the International Committee on Taxonomy of Viruses (ICTV), EP01 was classified into the T4-like viruses genus, *Myoviridae* family.

Different titers of EP01 were mixed with host bacteria and incubated for 4 h, the phage titers were determined by the double-layer plate methods. The results showed that the phage titers reached the highest at MOI of 0.001 and followed by 1, indicating that the optimal MOI of EP01 is 0.001, (Figure 2C). These results provided a reference for subsequent food experiments. One-step growth curve revealed that the latent period of EP01 was approximately 20 min (Figure 2D). The phage titer was then increased dramatically in the next 50 min. EP01 was under stable growth until 120 min subsequently and calculated to have a burst size of about 216 PFU/CFU.

For pH stability test, EP01 was relatively stable at pH ranging from 4 to 10 (Figure 2E). When exposed to strong acid or strong alkali (pH < 4 or pH > 10), phage titer decreased dramatically. Furthermore, EP01 was inactive when exposed to either pH 3 or pH 12. For thermal stability test, EP01 exhibited a high thermal tolerance as manifested by its stability at 30–60 °C. At 70 °C, phage titer decreased in the first 30 min, followed by increasing gradually in the next 30 min. Phage titer was undetectable after 60 min at 80 °C (Figure 2F).

### 3.3. Phage Tequatrovirus EP01 Genomic Analysis

**Figure 3:**
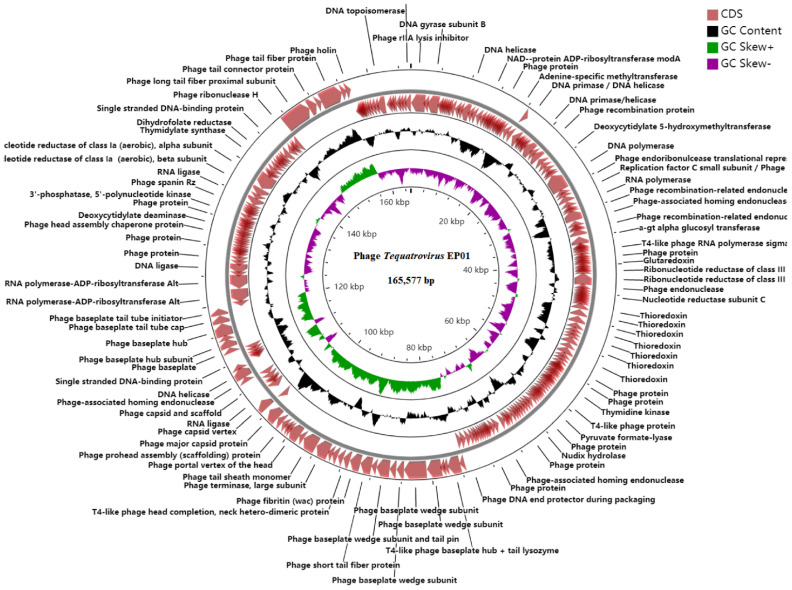
Comparative circular genome map of phage *Tequatrovirus* EP01. GC content is presented in black; positive and negative GC skew is indicated by green and purple.

EP01 has a double-stranded DNA genome consisting of 165,577 bp with a GC content of 35.43% (Figure 3). No genes associated with antibiotic resistance, toxins, and virulence factors were found in the genome, indicating the safety of EP01. EP01 was assigned to the T4−like viruses genus, *Myoviridae* family based on the morphology and genome sequence. A total of 257 CDSs were predicted in EP01 genome, of which 118 CDSs (45.91%) were predicted to encode functional proteins (Figure 3). Among 118 functional genes, 55 genes were responsible for DNA replication and repair; 45 genes were involved in structure and packaging; 8 genes were associated with host lysis; 6 genes were transcriptional regulators (Supplementary Table S2). 

**Figure 4:**
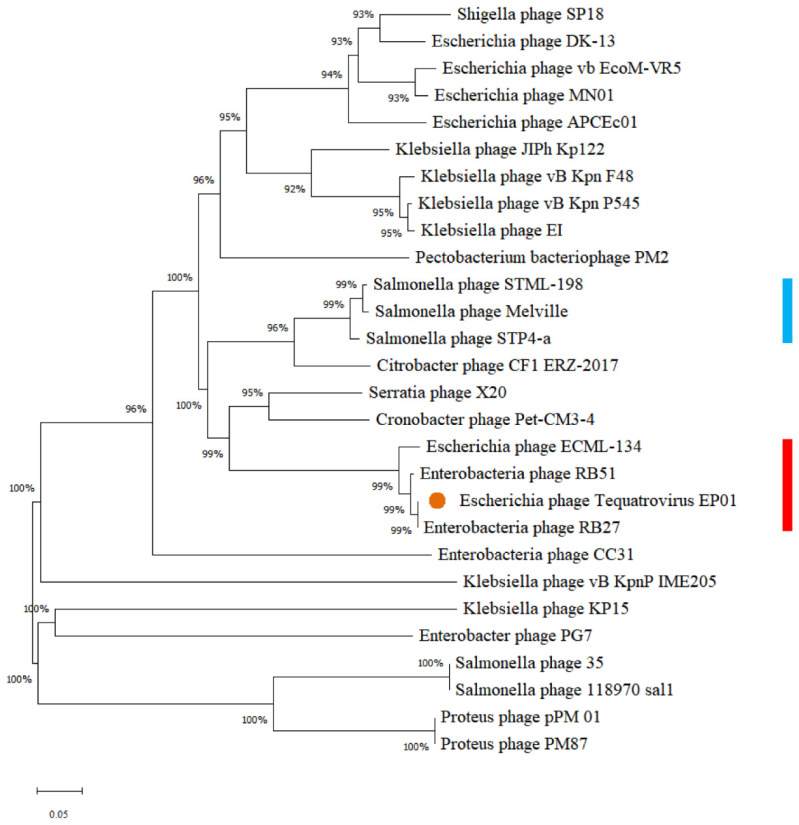
Phylogenetic analysis based on the sequences of major capsid proteins from 28 phages using MEGA X with 500 bootstraps. Orange dot locates the phage *Tequatrovirus* EP01. Red line indicates the *Tequatrovirus* cluster and blue *Gelderlandvirus* members.

**Figure 5:**
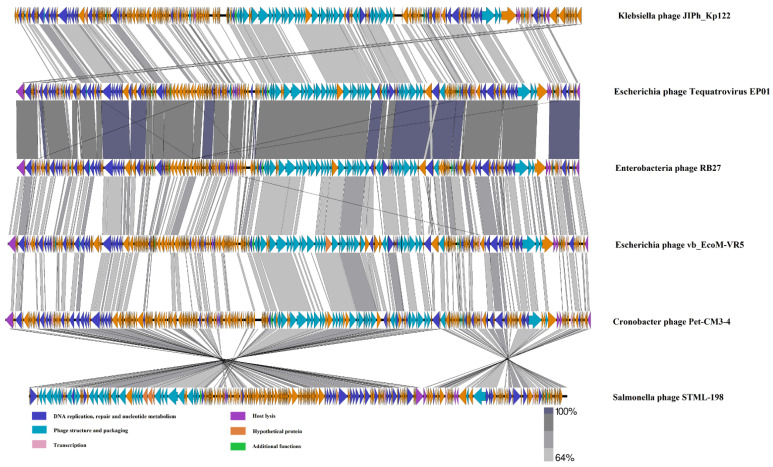
Comparative linear genome map of *Klebsiella* phage JIPh_Kp122, *Escherichia* phage *Tequatrovirus* EP01, *Enterobacteria* phage RB27, *Escherichia* phage vb_EcoM-VR5, *Cronobacter* phage Pet-CM3-4, and *Salmonella* phage STML-198 generated using Easyfig 2.2.5. Genes with different functions are denoted by different colors as shown in the legend. Similarity among genomes is depicted by grey lines based on BLASTN identity.

Phylogenetic analysis based on sequences of major capsid proteins showed that EP01 was located in the same branch with *Enterobacteria* phages RB27, RB51, and *Escherichia* phage ECML-134. These phages were grouped together in one clade (red), which represented they belong to the family *Myoviridae*, genus *Tequatrovirus* (Figure 4). A total of 142 conserved proteins are homologous (identity > 64 %) between EP01 (*Tequatrovirus*) and *Klebsiella* phage JIPh_Kp122 (*Jiaodavirus*) (Figure 5). Furthermore, 247 conserved proteins are extensively homologous (identity > 64 %) between EP01 (*Tequatrovirus*) and *Enterobacteria* phage RB27 (*Tequatrovirus*), including DNA primase, tail fibers, and capsid proteins (Figure 5).

### 3.4. Phage Tequatrovirus EP01 Could Inhibit the Growth of S. Enteritidis, E. coli O157:H7, E. coli O114:K90 (B90), and E. coli O142:K86 (B) in LB Broth

**Figure 6:**
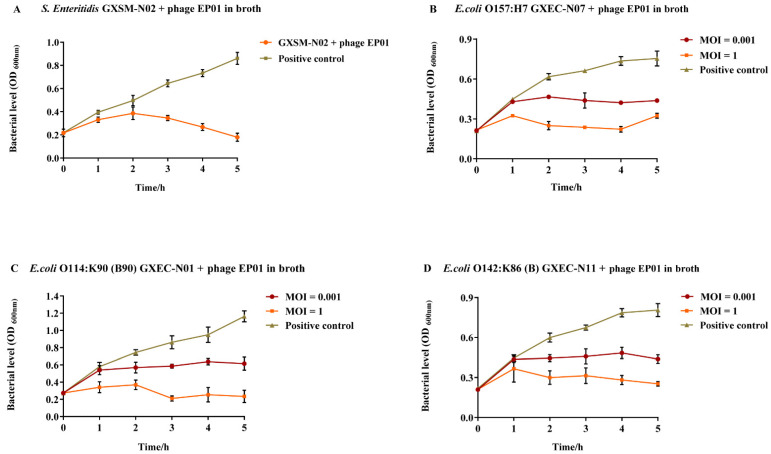
Inhibitory effect of phage *Tequatrovirus* EP01 on four pathogenic isolates in LB broth medium. (**A**) Inhibition of Salmonella Enteritidis GXSM-N02 growth. (**B**) Inhibition of *E. coli* O157:H7 GXEC-N07 growth. (**C**) Inhibition of *E. coli* O114:K90 (B90) GXEC-N01 growth. (**D**) Inhibition of *E. coli* O142:K86 (**B**) GXEC-N11 growth. Optical Density (OD) is defined as the optical density of a light-absorbing substance. The concentration of the bacterial cultures was measured by spectrophotometer at OD_600 nm_.

As shown in Figure 6, EP01 could persistently inhibit the growth of all the tested *Salmonella* and *E. coli* strains. In the positive control groups, the number of all the host strains obviously increased from 1 h to 5 h. After EP01 treatment, the viable counts of *S. Enteritidis* were slightly elevated within 2 h and then reduced significantly (*p* < 0.001) at 3–5 h compared to the positive control (Figure 6A). Similar results were also found in *E. coli* groups (Figure 6B–D). The results showed that at an MOI of 0.001, all the tested *E. coli* bacteria contents exhibited a slow growth in the first hour and then reached a stable level from 2 h to 5 h after phage treatment. At an MOI of 1, the viable counts of *E. coli* O157:H7, *E. coli* O114:K90 (B90), and *E. coli* O142:K86 (B) were increased slightly within 1 h and then reduced significantly to the initial level gradually with 2–5 h of phage treatment (*p* < 0.001). In general, the antibacterial effect of EP01 showed MOI dependence that the efficacy at the MOI of 1 is better than that at the MOI of 0.001.

### 3.5. Phage Tequatrovirus EP01 Could Control S. Enteritidis, E. coli O157:H7, E. coli O114:K90 (B90), and E. coli O142:K86 (B) Contamination on the Surface of Raw Meat and in Fresh Milk

#### 3.5.1. Application of Phage *Tequatrovirus* EP01 Controlling *S. Enteritidis*

**Figure 7:**
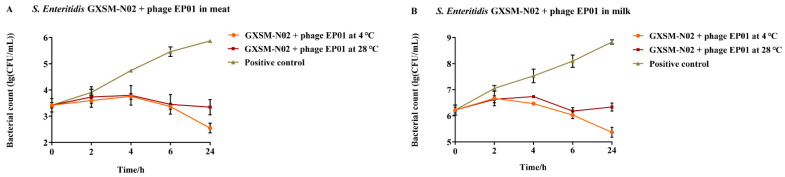
Antimicrobial effect of phage *Tequatrovirus* EP01 on the viability of *Salmonella Enteritidis* GXSM-N02 in different foods. (**A**) Effect of phage *Tequatrovirus* EP01 on the viability of *Salmonella Enteritidis* GXSM-N02 on raw meat stored at 4 °C and 28 °C. (**B**) Effect of phage *Tequatrovirus* EP01 on the viability of *Salmonella Enteritidis* GXSM-N02 on fresh milk stored at 4 °C and 28 °C. CFU stands for “colony forming units”. CFU /mL refers to the total number of bacterial communities per milliliter of sample.

The antimicrobial effects were closely associated with temperature that the efficacy of EP01 was shown to be higher at 4 °C than that at 28 °C (Figure 7). At 4 °C and 28 °C, the bacterial counts of *S. Enteritidis* GXSM-N02 exhibited slight rise in the first 4 h of incubation, and then decreased on the surface of foods. Treatment with EP01 significantly reduced viable bacteria of *S. Enteritidis* GXSM-N02 (*p* < 0.05) after incubation for 4, 6, 24 h (Figure 7A). The greatest reductions of 3.3 log_10_ CFU/cm^2^ (*p* < 0.001) and 2.5 log_10_ CFU/cm^2^ (*p* < 0.001) were observed following phage treatment for 24 h compared to positive control groups, respectively (Figure 7A). Similar effects were also found in milk; reductions in bacterial counts were observed after treatments with EP01 at all time points at 4 °C and 28 °C (Figure 7B). After 24 h incubation, greatest significant reductions of 3.5 log_10_ CFU/mL (*p* < 0.01) were observed at 4 °C whereas remarkable reductions of 2.5 log_10_ CFU/mL (*p* < 0.001) were observed at 28 °C (Figure 7B).

#### 3.5.2. Application of Phage *Tequatrovirus* EP01 Controlling *E. coli* O157:H7

**Figure 8:**
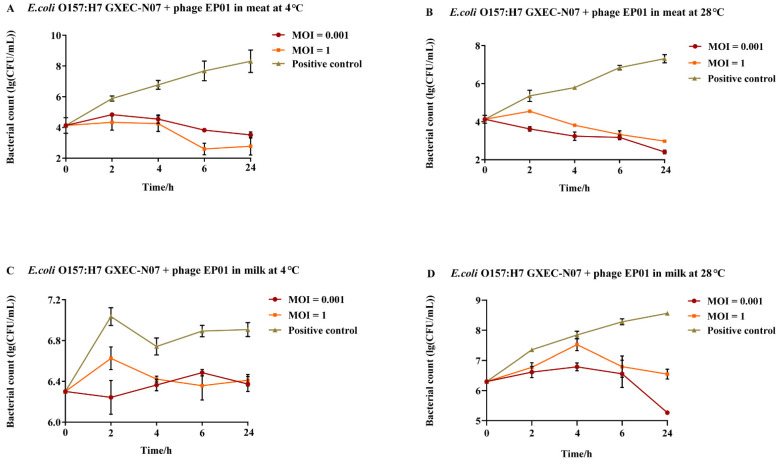
Antimicrobial effect of phage *Tequatrovirus* EP01 on the viability of *E. coli* O157:H7 GXEC-N07 in different foods. (**A**,**B**) Effect of phage *Tequatrovirus* EP01 on the viability of *E. coli* O157:H7 GXEC-N07 on raw meat stored at 4 °C and 28 °C. (**C**,**D**) Effect of phage *Tequatrovirus* EP01 on the viability of *E. coli* O157:H7 GXEC-N07 on fresh milk stored at 4 °C and 28 °C.

EP01 presented a considerable inhibition on the growth of *E. coli* O157:H7 GXEC-N07 on the surface of meat (5.53 log_10_ CFU/cm^2^) and in milk (3.3 log_10_ CFU/mL) compared to positive control (*p* < 0.001, Figure 8). In *E. coli* O157:H7 contaminated meat, treatment with EP01 at MOIs of 0.001 and 1 resulted in significant reductions of bacterial number after 4, 6, 24 h of incubation at 4 °C and 28 °C (*p* < 0.05, Figure 8A,B). At 4 °C, EP01 addition at an MOI of 0.001 presented a lower efficiency than that at an MOI of 1 (Figure 8A), whereas at 28 °C showed the contrary result (Figure 8B). In milk, at 4 °C, phage addition at an MOI of 0.001 significantly reduced viable bacteria at 2, 24 h (*p* < 0.05) whereas at an MOI of 1, significant reductions were observed at 2, 6, 24 h (*p* < 0.05, Figure 8C). At 28 °C, phage treatment at an MOI of 1 showed a stronger antibacterial effect than that at MOI of 0.001. Significant reductions were observed after incubation for 6, 24 h at MOIs of 0.001 and 1 (*p* < 0.05, Figure 8D).

#### 3.5.3. Application of Phage *Tequatrovirus* EP01 Controlling *E. coli* O114:K90 (B90)

**Figure 9:**
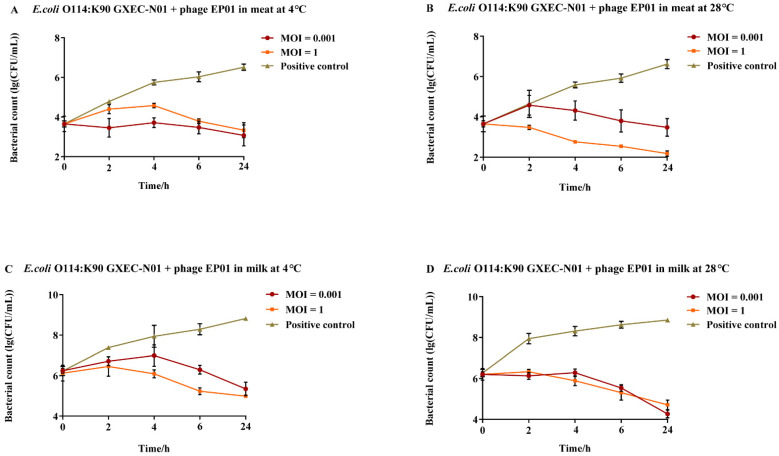
Antimicrobial effect of phage *Tequatrovirus* EP01 on the viability of *E. coli* O114:K90 (B90) GXEC-N01 in different foods. (**A**,**B**) Effect of phage *Tequatrovirus* EP01 on the viability of *E. coli* O157:H7 GXEC-N01 on raw meat stored at 4 °C and 28 °C. (**C**,**D**) Effect of phage *Tequatrovirus* EP01 on the viability of *E. coli* O157:H7 GXEC-N01 on fresh milk stored at 4 °C and 28 °C.

On the contaminated surface of meat and milk, EP01 could reduce the viable counts of *E. coli* O114:K90 (B90) GXEC-N01 to below the initial level (2.18 log_10_ CFU/cm^2^, 4.26 log_10_ CFU/mL). In meat, addition of EP01 at MOIs of 0.001 and 1 significantly reduced the bacterial counts at 4 °C and 28 °C after incubation for 6 h and 24 h, respectively (*p* < 0.01, Figure 9A,B). At 4 °C, the antibacterial effect showed no remarkable differences between phage treatment at MOIs of 0.001 and 1 (Figure 9A). At 28 °C, the efficiency of two phage treatment groups at different MOIs began to present a significant difference after incubation for 2 h (*p* < 0.01), and phage treatment at an MOI of 0.001 exhibited a lower efficiency than that at an MOI of 1 (Figure 9A). In milk, treatment with EP01 at an MOI of 0.001 significantly reduced viable bacteria at 6, 24 h (*p* < 0.01) at 4 °C compared to phage free controls, whereas at an MOI of 1, remarkable reductions were observed at 4, 6, and 24 h (*p* < 0.05, Figure 9C). At 28 °C, significant reductions in viable bacteria were observed at MOIs of 0.001 and 1 after incubation for 4, 6, 24 h (Figure 9D).

#### 3.5.4. Application of Phage *Tequatrovirus* EP01 Controlling *E. coli* O142:K86 (B)

**Figure 10:**
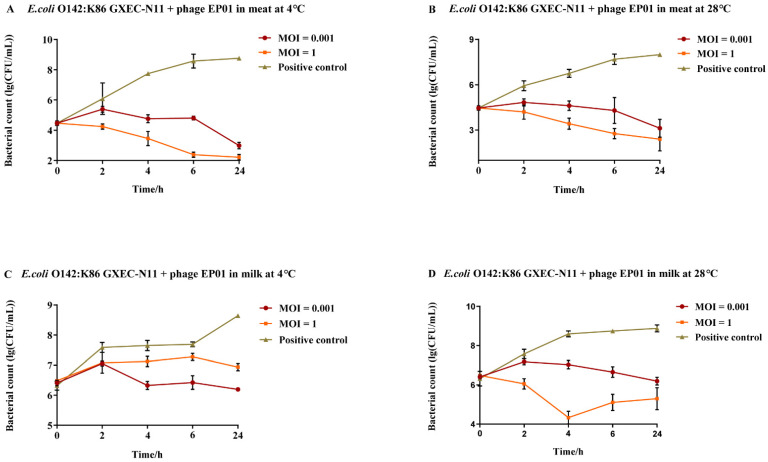
Antimicrobial effect of phage *Tequatrovirus* EP01 on the viability of *E. coli* O142:K86 (B) GXEC-N11 in different foods. (**A**,**B**) Effect of phage *Tequatrovirus* EP01 on the viability of *E. coli* O142:K86 (B) GXEC-N11 on raw meat stored at 4 °C and 28 °C. (**C**,**D**) Effect of phage *Tequatrovirus* EP01 on the viability of *E. coli* O142:K86 (B) GXEC-N11 on fresh milk stored at 4 °C and 28 °C.

As shown in Figure 10, EP01 treatment could cause reductions of 6.55 log_10_ CFU/cm^2^ and 4.26 log_10_ CFU/mL in the viable counts of *E. coli* O142:K86 (B) GXEC-N11 on the surface of meat and milk compared to phage-free groups, respectively (*p* < 0.001). In meat, the antibacterial effect of EP01 showed dosage dependence. At MOIs of 0.001 and 1, significant reductions were observed after incubation for 4, 6, 24 h at 4 °C and 28 °C (*p* < 0.05, Figure 10A,B). In contaminated milk at 4 °C, significant reductions were observed following application of EP01 at MOIs of 0.001 and 1 after 24 h of incubation (Figure 10C,D). Moreover, the antibacterial efficiency at an MOI of 0.001 was shown to be higher than that at an MOI of 1, and significant differences began to appear between treatment groups of two different MOIs after incubation of 4 h (*p* < 0.05, Figure 10C). At 28 °C, addition of EP01 at MOIs of 0.001 and 1 significantly reduced the viable counts at 4, 6, 24 h (*p* < 0.05, Figure 10D). The greatest reductions of 4.3 log_10_ CFU/mL (*p* < 0.001) were observed at 4 h with phage treatment at an MOI of 1 (Figure 10D).

## 4. Discussion

One of the main limitations of the widespread application of phages is the narrow host range. Generally, the sensitivity rate of a single phage against bacterial strains within a species usually ranges from a few percent to the teens. It is hard to obtain phages that could effectively lyse all the strains of a species or multiple bacterial species. Phage cocktails and polyvalent broad-spectrum phages are proposed to solve this problem. However, combination of multiple phages may produce antagonistic effect and lead to complex pharmacological phenomena that affect the bacteriostatic effect. Therefore, the isolation and development of polyvalent broad-spectrum phages is particularly important. Herein, we isolated a T4 phage named *Tequatrovirus* EP01. Spot tests and antibacterial assay in LB broth certified the cracking ability of EP01 against 32 bacterial strains within species of *Salmonella* and *Escherichia coli*.

Phages bind to specific receptors on the surface of the bacteria using their receptor-binding proteins (RBPs) including tails fibers proteins or tail spike proteins. EP01 encodes 12 coding DNA sequences (CDSs) associated with RBPs. There are 11 CDSs encoding tail fiber proteins and 1 CDS encoding receptor-recognizing protein. As the binding of the RBPs to the O-antigen is highly specific, phages encode diverse RBPs to match the O-antigen diversity [26,27]. These RBPs allow EP01 to bind to diverse O-antigens of Lipopolysaccharide (LPS), which enable EP01 to recognize different receptors. This may be one of the most important reasons why EP01 could lyse multiple host bacteria of multiple serotypes.

Previous studies have demonstrated that the interaction between the gp37 protein of T4 phages long tail fibers and outer membrane protein C (OmpC) mainly determines their host specificity [28]. CDS 239 encodes gp37 protein in the EP01 genome. Based on blastn result of NCBI database, CDS 239 shares high homology with corresponding genes of many *Escherichia coli* phages (78.13% < percent identities < 94.29%) and a *Salmonella* phage (percent identities is 85.51%). Similarly, receptor-recognizing protein encoded by CDS 240 shares 95.29% homology with a *Salmonella* phage and high homology (74.45% < percent identities < 96.92%) with *Escherichia coli* phages. These above points perhaps explain why EP01 could both infect *Escherichia coli* and *Salmonella*. The specific interaction mechanism between EP01 and their hosts needs to be elucidated by further experiments.

EP01 shares 75.35% homology with *Klebsiella* phage JIPh_Kp122 according to blastn result of NCBI database. There are 142 homologous conserved proteins (identity > 64%) between EP01 and *Klebsiella* phage JIPh_Kp122, including tail fibers proteins, DNA helicase, DNA primase, DNA polymerase. These points indicate that EP01 could probably lyse *Klebsiella pneumoniae* besides *Escherichia coli* and *Salmonella*. EP01 is expected to be a polyvalent phage that might lyse three species of bacteria.

It has been previously reported that several phages remained active under the high temperature of 60 °C and pH ranging from 4 to 10 [29,30]. In line with the previous studies, EP01 was relatively stable under a wide range of pH from 4 to 10. That might enable EP01 to have longer shelf life in food environment and allow the application of EP01 in foods at different pH levels. Besides, EP01 presented a considerable thermal stability as the phage titer was detectable up to 80 °C for 1 h, which is more tolerant than many other previously reported phages. This indicated that EP01 could be used in combination with pasteurization to sterilize dairy products.

Foodborne diseases caused by microbial contamination frequently occur all around the world. Phages as vital antibacterial agents play an important role in the food application field with its advantages of safety, convenience, easy preparation, and low cost. The success of phage biological control mainly depends on food matrices, temperature, and phage dosage. In this study, we selected two animal-origin foods (meat and milk) as matrices and studied the biological control effects of polyvalent broad-spectrum phage EP01 against *Salmonella* and *Escherichia coli* in these contaminated foods.

At different temperatures (4 °C and 28 °C), the viable counts of *Salmonella Enteritidis* were significantly reduced (*p* < 0.001) on the surface of meat (3.3 log_10_ CFU/cm^2^) and milk (3.5 log_10_ CFU/mL). We found that temperature largely influenced the effectiveness of EP01. In *Salmonella Enteritidis* contaminated meat and milk, the bacteriostatic effects at 4 °C showed better than that at 28 °C after 6 h of incubation with significant difference (*p* < 0.05). Furthermore, the regrowth of host bacteria was observed after 6 h. The results were contrary to previous studies on the efficacy of phage LPST10 in sausage at 4 °C and 28 °C [11], indicating that EP01 had good bacteriostatic effect even under refrigeration temperature. We guess these results might be because (ⅰ) the emergence of phage-resistant strains after 6 h inhibited the growth of EP01. Temperature affected the metabolic rate of bacterial cells and the growth velocity of phage-resistant strains was lower when refrigerated at 4 °C; (ⅱ) EP01 can keep highly active for a long time under refrigerated temperature, so that it can remove germs from the surface of meat and milk effectively; (ⅲ) storage at 4 °C could prevent bacteria regrowth after phage treatment, and the reduction in bacterial counts could persist for a few days under low temperature [11]; (ⅳ) the life cycle and replication of phages depend on metabolic function of host bacteria. The activity of *Salmonella Enteritidis* was relatively lower at 4 °C than that at 28 °C, so the growth of EP01 was inhibited; (ⅴ) phage particles with high densities absorb onto the bacterial cell surface, which may result in cell destruction, low cell envelope stability, and even cell death without phages formation. EP01 might lead to the above phenomenon called lysis from without with the help of tail lysozyme (CDS 119, CDS 143) and the baseplate hub subunit (CDS 180, CDS 181, CDS 183, CDS 185).

The bacteriostatic effects of EP01 were observed to present better when applied to phage lysates with higher titers. The addition of phages of different concentrations (10^7^ PFU/mL, 10^10^ PFU/mL) reduced the viable counts of *E. coli* O142:K86 (B) (2.69 log_10_ CFU/mL and 4.26 log_10_ CFU/mL, *p* < 0.001) in contaminated milk at 28 °C. In meat, significant reductions of *E. coli* O142:K86 (B) counts (4.87 log_10_ CFU/cm^2^ and 5.6 log_10_ CFU/cm^2^, *p* < 0.001) were observed with EP01 treatment (10^6^ PFU/mL, 10^9^ PFU/mL). Similar results were reported by Zhang et al., suggesting that addition of high dosage of EP01 could remove bacteria contamination from the surface of foods with better efficiency. This might be due to the fact that high dosage of EP01 induced more collisions between phage particles and bacterial cells on the surface of foods. Furthermore, that enables EP01 to infect host bacteria with higher probabilities. Besides, phage particles of high densities largely reduced the risk of immobilizing by food matrices.

Although EP01 was proved to be effective for removing pathogen contamination from the surface of meat and milk, there are still some limitations: (ⅰ) EP01 could only target specific hosts (*Salmonella* and *Escherichia coli*) when applied in foods; (ⅱ) EP01 could only reduce the viable counts of hosts to below the initial level at most; it was not able to completely remove bacteria from the surface of meat and milk; (ⅲ) phage-resistant strains may develop during the food application possess and affect the antimicrobial effect; (ⅳ) EP01 particles were easily immobilized by food matrices, which affected the abilities of particles diffusion and infecting pathogens. In view of the above problems, we put forward several solutions. Combined utilization of EP01 and other phages could target more species of bacteria and prevent the emergence of phage-resistant strains. Moreover, multiple combinations of phages and other food-grade antimicrobials or high concentration of phages could be used to improve the antibacterial effectiveness.

## 5. Conclusions

Our study described a polyvalent broad-spectrum phage EP01 isolated from pig farm sewage. Biological characterization experiments and genomic sequence demonstrated that EP01 was relatively stable at different temperature and pH levels and safe. Besides, EP01 could inhibit the growth of *Salmonella* and *Escherichia coli* in LB broth and in two different foods. This experiment indicated that polyvalent broad-spectrum phages have the potential of controlling foodborne pathogens and could be considered as natural alternative biocontrol agents in the food industry.

## Data Availability

The data presented in this study are available on request from the corresponding author.

## Supplementary Materials

The following supporting information can be downloaded at: https://www.mdpi.com/article/10.3390/v14020286/s1, Figure S1: Morphology on double-layer agar plates of six isolated phages. (A) The plague of phage EP01. (B) The plague of phage EP02. (C) The plague of phage EP03. (D) The plague of phage EP04. (E) The plague of phage EP05. (F) The plague of phage EP06, Figure S2: The plaque phenotypes of phage EP01 performed on different host strains. (A) The plaque on *E. coli* O114:K90 (B90) GXEC-N01. (B) The plaque on *E. coli* O157:H7 GXEC-N07. (C) The plaque on *E. coli* O142:K86 (B) GXEC-N11. (D) The spot on *Salmonella Enteritidis* GXSM-N02. “+”, phage EP01 lysate; “−”, negative control, Table S1: Bacterial strains information, Table S2: CDS function prediction of phage EP01.
