# Supplementary material for: A Polyvalent Broad-Spectrum Escherichia Phage Tequatrovirus EP01 Capable of Controlling Salmonella and Escherichia coli Contamination in Foods
Source: Viruses. 2022 Jan 29;14(2):286. doi: 10.3390/v14020286 (PMC8877722; doi:10.3390/v14020286)
Supplement: Supplementary file 1 [file viruses-14-00286-s001.zip › viruses-1536839-supplementary.pdf]

viruses

Supplementary Material

A Polyvalent Broad-Spectrum *Escherichia* Phage *Tequatrovirus* EP01 Capable of Controlling *Salmonella* and *Escherichia coli* Contamination in Foods

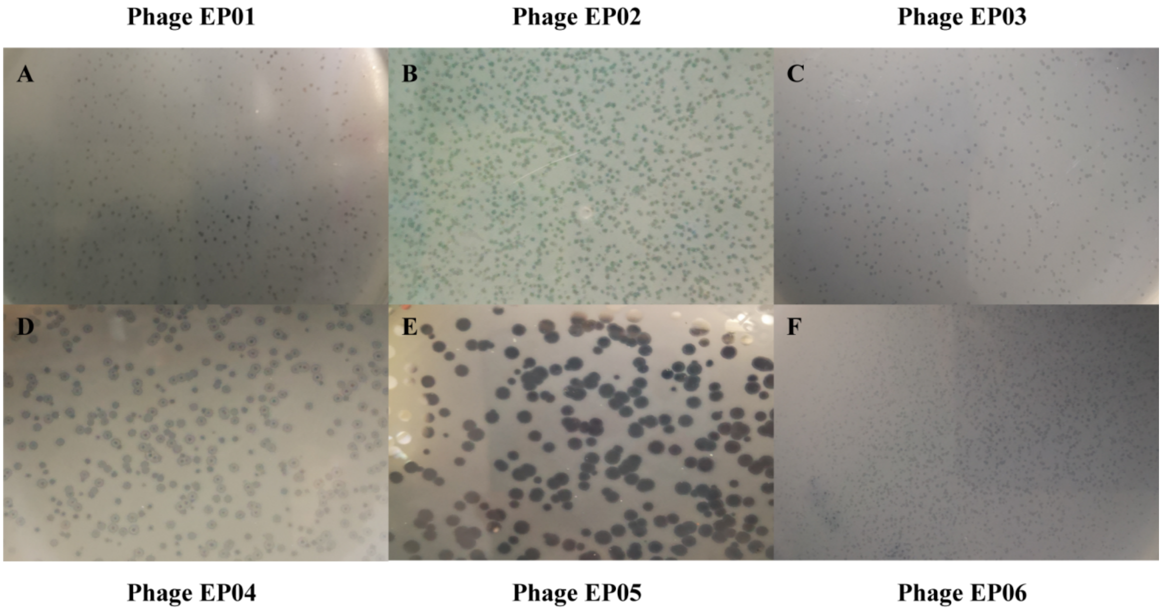

Figure S1. Morphology on double-layer agar plates of six isolated phages. (**A**) The plaque of phage EP01 performed on the host strain *E. coli* O114:K90 (B90) GXEC-N01. (**B**) The plaque of phage EP02 performed on the host strain *E. coli* O127a:K63(B8) GXEC-N12. (**C**) The plaque of phage EP03 performed on the host strain *E. coli* O127a:K63(B8) GXEC-C01. (**D**) The plaque of phage EP04 performed on the host strain *E. coli* O8:K88 CVCC1527. (**E**) The plaque of phage EP05 performed on the host strain *E. coli* O157:H7 CVCC4050. (**F**) The plague of phage EP06 performed on the host strain *E. coli* O127a:K63(B8) GDEC-F04.

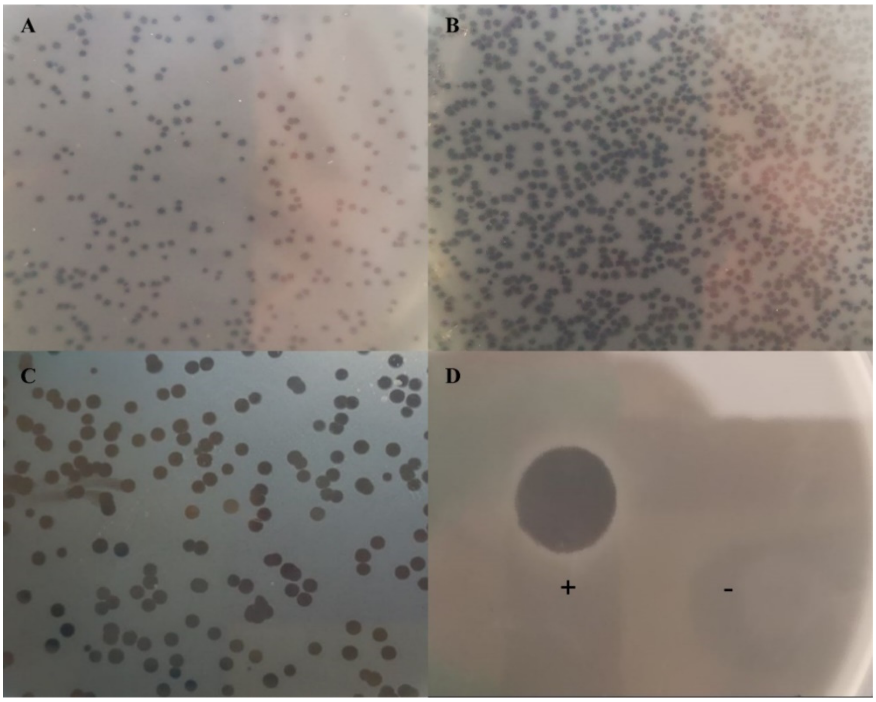

Figure S2. The plaque phenotypes of phage EP01 performed on different host strains. (**A**) The plaque on *E. coli* O114:K90 (B90) GXEC-N01. (**B**) The plaque on *E. coli* O157:H7 GXEC-N07. (**C**) The plaque on *E. coli* O142:K86 (B) GXEC-N11. (**D**) The spot on *Salmonella Enteritidis* GXSM-N02. "+", phage EP01 lysate; "-", negative control.

Table S1. Bacterial strains information.

Strains	Genus	Place	Source	Serotype/ Capsular type
GXEC-N01	*Escherichia coli*	Guangxi	Pig	O114:K90(B90)
GXEC-N02	*Escherichia coli*	Guangxi	Human	Undetected
GXEC-N03	*Escherichia coli*	Guangxi	Human	Undetected
GXEC-N04	*Escherichia coli*	Guangxi	Pig	O126:K71(B16)
GXEC-N05	*Escherichia coli*	Guangxi	Pig	O26:K60(B6)
GXEC-N06	*Escherichia coli*	Guangxi	Pig	O126:K71(B16)
GXEC-N07	*Escherichia coli*	Guangxi	Pig	O157:H7
GXEC-N08	*Escherichia coli*	Guangxi	Pig	Undetected
GXEC-N09	*Escherichia coli*	Guangxi	Pig	Undetected
GXEC-N10	*Escherichia coli*	Guangxi	Pig	Undetected
GXEC-N11	*Escherichia coli*	Guangxi	Pig	O142:K86(B)
GXEC-N12	*Escherichia coli*	Guangxi	Human	O127a:K63(B8)
GXEC-N13	*Escherichia coli*	Guangxi	Pig	Undetected
GXEC-N14	*Escherichia coli*	Guangxi	Pig	Undetected
GXEC-N15	*Escherichia coli*	Guangxi	Pig	Undetected
GXEC-N16	*Escherichia coli*	Guangxi	Pig	Undetected
GXEC-N17	*Escherichia coli*	Guangxi	Pig	Undetected
GXEC-N18	*Escherichia coli*	Guangxi	Pig	Undetected
GXEC-N19	*Escherichia coli*	Guangxi	Pig	Undetected
GXEC-N20	*Escherichia coli*	Guangxi	Pig	Undetected
GXEC-N21	*Escherichia coli*	Guangxi	Pig	Undetected
GXEC-N22	*Escherichia coli*	Guangxi	Pig	Undetected
GXEC-N23	*Escherichia coli*	Guangxi	Pig	Undetected
GXEC-N24	*Escherichia coli*	Guangxi	Pig	Undetected
GXEC-N25	*Escherichia coli*	Guangxi	Pig	Undetected
GXEC-N26	*Escherichia coli*	Guangxi	Pig	Undetected
GXEC-N27	*Escherichia coli*	Guangxi	Human	Undetected

GXEC-B01	*Escherichia coli*	Guangxi	Pet	Undetected
GXEC-B02	*Escherichia coli*	Guangxi	Pet	Undetected
GXEC-C01	*Escherichia coli*	Guangxi	Bovine	O127a:K63(B8)
GDEC-F01	*Escherichia coli*	Guangdong	Pig	Undetected
GDEC-F02	*Escherichia coli*	Guangdong	Pig	Undetected
GDEC-F03	*Escherichia coli*	Guangdong	Environment	Undetected
GDEC-F04	*Escherichia coli*	Guangdong	Environment	O127a:K63(B8)
GDEC-F05	*Escherichia coli*	Guangdong	Environment	O127a:K63(B8)
GDEC-F06	*Escherichia coli*	Guangdong	Environment	O127a:K63(B8)
GDEC-F07	*Escherichia coli*	Guangdong	Environment	O111:K58(B4)
GDEC-F08	*Escherichia coli*	Guangdong	Environment	Undetected
GDEC-F09	*Escherichia coli*	Guangdong	Environment	Undetected
GDEC-F10	*Escherichia coli*	Guangdong	Environment	Undetected
GDEC-F11	*Escherichia coli*	Guangdong	Pig	Undetected
GDEC-F12	*Escherichia coli*	Guangdong	Pig	Undetected
GDEC-F13	*Escherichia coli*	Guangdong	Pig	Undetected
GDEC-F14	*Escherichia coli*	Guangdong	Pig	Undetected
GDEC-F15	*Escherichia coli*	Guangdong	Environment	Undetected
SCEC-Z01	*Escherichia coli*	Sichuan	Avian	Undetected
SCEC-Z02	*Escherichia coli*	Sichuan	Avian	Undetected
SCEC-Z03	*Escherichia coli*	Sichuan	Avian	Undetected
SCEC-Z04	*Escherichia coli*	Sichuan	Avian	Undetected
SCEC-Z05	*Escherichia coli*	Sichuan	Avian	Undetected
SCEC-Z06	*Escherichia coli*	Sichuan	Avian	Undetected
SCEC-Z07	*Escherichia coli*	Sichuan	Avian	O157
SCEC-Z08	*Escherichia coli*	Sichuan	Avian	Undetected
SCEC-Z09	*Escherichia coli*	Sichuan	Avian	Undetected
SCEC-Z10	*Escherichia coli*	Sichuan	Avian	Undetected
HBEC-J01	*Escherichia coli*	Hubei	Pet	Undetected
SXEC-X01	*Escherichia coli*	Shanxi	Pet	Undetected
GXKP-J05	*Klebsiella pneumoniae*	Guangxi	Pig	K20
GXKP-B2	*Klebsiella pneumoniae*	Guangxi	Pig	K1
GXKP-D4	*Klebsiella pneumoniae*	Guangxi	Dog	K2
GXKP-F9	*Klebsiella pneumoniae*	Guangxi	Pig	K20
GXKP-H4	*Klebsiella pneumoniae*	Guangxi	Pig	K1
GXKP-H6	*Klebsiella pneumoniae*	Guangxi	Pig	K2
SCKP-15	*Klebsiella pneumoniae*	Sichuan	Avian	K57
SCKP-38	*Klebsiella pneumoniae*	Sichuan	Avian	K20
GDKP-7	*Klebsiella pneumoniae*	Guangdong	Pig	K1
GDKP-28-4	*Klebsiella pneumoniae*	Guangdong	Pig	K20
GXSM-N01	*Salmonella enteria serovar Typhimurium*	Guangxi	Pig	Undetected
GXSM-N02	*Salmonella enteria serovar Typhimurium*	Guangxi	Pig	Undetected
GXPM-N01	*Proteus mirabilis*	Guangxi	Pig	Undetected
GXPM-N02	*Proteus mirabilis*	Guangxi	Pig	Undetected
SCPA-Z01	*Pseudomonas aeruginosa*	Sichuan	Cat	Undetected
CVCC1527	*Escherichia coli*	China Veterinary Culture Collection Center (CVCC)	Pig	O8:K88
CVCC4050	*Escherichia coli*	China Veterinary Culture Collection Center (CVCC)		O157:H7
CVCC1806	*Salmonella enteria serovar Typhimurium*	China Veterinary Culture Collection Center (CVCC)	Avian	Undetected
CVCC3384	*Salmonella typhimurium*	China Veterinary Culture Collection Center (CVCC)		Undetected

Note: Undetected means the strain was not belong to these serotypes (O157:H7, O157, O114:K90(B90), O126:K71(B16), O26:K60(B6), O142:K86(B), O127a:K63(B8), O111:K58(B4)) or these capsular types (K1, K2, K5, K20, K54, K57).

Table S2. CDS function prediction of phage EP01.

CDS	Hit_name	Hit_description	Length(aa)	Identity (%)	value
1	YP_009148452.1	putative protector from prophage-induced early lysis [Escherichia phage HY01]	725	99.03	0.00E+00
2	YP_009167813.1	hypothetical protein [Escherichia phage AR1]	67	98.51	3.00E-29
3	ANH49860.1	DNA gyrase subunit B [Escherichia phage PE37]	605	100.00	0.00E+00
4	APU02635.1	hypothetical protein EPG_085 [Escherichia phage YUEEL01]	86	98.84	2.10E-43
5	YP_007004387.1	hypothetical protein [Escherichia phage ime09]	123	97.56	5.00E-62
6	YP_002854342.1	hypothetical protein [Enterobacteria phage RB14]	58	100.00	9.10E-30
7	YP_009030893.1	mRNA metabolism modulator [Escherichia phage vB_EcoM_112]	139	97.12	2.50E-73
8	YP_002854344.1	modifier of suppressor T4 tRNAs [Enterobacteria phage RB14]	71	100.00	1.00E-35
9	YP_009030895.1	hypothetical protein e112_011 [Escherichia phage vB_EcoM_112]	89	100.00	5.30E-45
10	YP_009288377.1	transcriptional regulator [Shigella phage SHBML-50-1]	163	97.55	1.70E-86
11	YP_007004396.1	exonuclease A [Escherichia phage ime09]	227	99.56	3.80E-132
12	YP_002854350.1	hypothetical protein [Enterobacteria phage RB14]	81	95.00	6.50E-34
13	YP_006986566.1	DNA helicase [Escherichia phage vB_EcoM_ACG-C40]	439	99.54	4.30E-257
14	YP_007004400.1	hypothetical protein [Escherichia phage ime09]	103	100.00	2.50E-54
15	YP_009148469.1	putative anti sigma factor [Escherichia phage HY01]	248	100.00	7.60E-134
16	YP_009148470.1	putative ADP-ribosylase [Escherichia phage HY01]	200	99.50	7.30E-111
17	APU02649.1	ADP-ribosylase [Escherichia phage YUEEL01]	207	99.03	8.00E-113
18	YP_007004404.1	hypothetical protein [Escherichia phage ime09]	60	98.33	1.80E-28
19	YP_004414922.1	hypothetical protein [Shigella phage Shfl2]	156	98.72	7.70E-84
20	YP_004414923.1	hypothetical protein [Shigella phage Shfl2]	54	98.15	2.20E-22
21	YP_802965.1	postulated decoy of host sigma32 [Enterobacteria phage RB32]	67	97.01	8.00E-30
22	YP_009148475.1	putative transcription modulator under heat shock [Escherichia phage HY01]	161	95.65	3.60E-84
23	YP_009148476.1	hypothetical protein [Escherichia phage HY01]	113	96.46	1.80E-58
24	YP_802969.1	hypothetical protein [Enterobacteria phage RB32]	70	98.57	5.40E-37

25	YP_009102235.1	small outer capsid protein [Enterobacteria phage RB27]	85	100.00	9.50E-44
26	YP_009180802.1	hypothetical protein [Escherichia phage slur14]	69	98.55	5.00E-35
27	YP_009102505.1	hypothetical protein [Escherichia phage ECML-134]	64	100.00	3.60E-27
28	YP_009030637.1	dCTP pyrophosphatase [Escherichia phage vB_EcoM_112]	172	99.42	1.40E-94
29	YP_009277673.1	adenine-specific methyltransferase [Shigella phage SHFML-11]	259	98.84	7.40E-148
30	YP_009030639.1	hypothetical protein [Escherichia phage vB_EcoM_112]	186	97.85	1.30E-101
31	YP_009098418.1	hypothetical protein [Enterobacteria phage RB3]	66	95.45	3.10E-26
32	YP_009148481.1	putative DNA primase [Escherichia phage HY01]	342	99.71	3.30E-204
33	YP_009277677.1	hypothetical protein [Shigella phage SHFML-11]	54	100.00	3.20E-21
34	YP_009110856.1	spackle periplasmic protein [Shigella phage pSs-1]	97	97.94	7.20E-48
35	YP_003934666.1	hypothetical protein [Shigella phage SP18]	84	54.88	7.20E-20
36	YP_004414939.1	hypothetical protein [Shigella phage Shfl2]	73	100.00	1.40E-35
37	YP_002854373.1	discriminator of mRNA degradation [Enterobacteria phage RB14]	60	98.33	1.10E-27
38	YP_009148487.1	putative DNA primase-helicase subunit [Escherichia phage HY01]	475	99.79	9.00E-269
39	YP_009148488.1	putative head vertex assembly chaperone [Escherichia phage HY01]	112	100.00	9.20E-55
40	YP_004414943.1	putative UvsX recA-like recombination protein [Shigella phage Shfl2]	393	100.00	2.90E-220
41	YP_007004427.1	beta-glucosyl-HMC-alpha-glucosyl-transferase [Escherichia phage ime09]	280	99.64	3.90E-163
42	YP_009277686.1	hypothetical protein [Shigella phage SHFML-11]	213	100.00	7.00E-128
43	YP_009277687.1	deoxycytidylate 5-hydroxymethyltransferase [Shigella phage SHFML-11]	246	98.78	1.70E-146
44	YP_009098431.1	immunity to superinfection membrane protein [Enterobacteria phage RB3]	83	98.80	3.80E-37
45	YP_007004788.1	hypothetical protein [Escherichia phage wV7]	126	98.41	3.10E-67
46	YP_009148495.1	putative DNA polymerase [Escherichia phage HY01]	898	99.89	0.00E+00
47	YP_009281390.1	translational repressor protein [Escherichia phage UFV-AREG1]	122	100.00	7.40E-66
48	YP_009148498.1	putative clamp-loader subunit [Escherichia phage HY01]	187	99.47	4.40E-102
49	YP_007004435.1	DNA polymerse clamp loader subunit [Escherichia phage ime09]	319	100.00	8.70E-183
50	YP_007004436.1	sliding clamp DNA polymerase accessory protein [Escherichia phage ime09]	228	100.00	5.50E-123

51	YP_004414954.1	putative RNA polymerase binding protein [Shigella phage Shfl2]	129	99.22	4.20E-67
52	YP_007004438.1	hypothetical protein [Escherichia phage ime09]	62	100.00	4.80E-29
53	YP_009102529.1	hypothetical protein [Escherichia phage ECML-134]	560	99.64	0.00E+00
54	YP_009277698.1	homing endonuclease [Shigella phage SHFML-11]	284	99.65	3.60E-156
55	YP_009281397.1	hypothetical protein [Escherichia phage UFV-AREG1]	68	100.00	7.90E-33
56	YP_004414958.1	hypothetical protein [Shigella phage Shfl2]	87	98.85	2.10E-46
57	YP_009284129.1	hypothetical protein [Escherichia phage HY03]	339	99.12	7.50E-201
58	YP_004414960.1	putative alpha-glucosyl-transferase [Shigella phage Shfl2]	400	100.00	8.20E-239
59	YP_803003.1	hypothetical protein [Enterobacteria phage RB32]	57	98.25	1.10E-24
60	NP_049676.1	hypothetical protein [Enterobacteria phage T4]	67	98.51	5.90E-33
61	YP_009197389.1	hypothetical protein [Escherichia phage slur07]	105	99.05	7.10E-49
62	YP_004414964.1	hypothetical protein [Shigella phage Shfl2]	72	98.39	5.60E-29
63	YP_009030673.1	sigma factor [Escherichia phage vB_EcoM_112]	185	100.00	8.00E-104
64	ADJ39784.1	hypothetical protein [Enterobacteria phage T4T]	71	98.59	1.30E-33
65	NP_049681.1	hypothetical protein [Enterobacteria phage T4]	108	100.00	8.30E-53
66	YP_009288438.1	hypothetical protein [Shigella phage SHBML-50-1]	66	98.48	4.90E-32
67	YP_009102545.1	hypothetical protein [Escherichia phage ECML-134]	97	100.00	1.00E-49
68	YP_007004455.1	hypothetical protein [Escherichia phage ime09]	58	98.28	2.10E-26
69	YP_009281413.1	glutaredoxin [Escherichia phage UFV-AREG1]	107	100.00	1.20E-56
70	YP_009210264.1	hypothetical product [Escherichia phage slur02]	87	96.55	1.00E-45
71	YP_002854411.1	hypothetical protein [Enterobacteria phage RB14]	71	100.00	1.90E-29
72	YP_009110895.1	gp88 [Shigella phage pSs-1]	156	98.72	1.10E-95
73	YP_007004460.1	ribonucleotide reductase of class III large subunit [Escherichia phage ime09]	605	99.67	0.00E+00
74	YP_803019.1	recombinase endonuclease VII [Enterobacteria phage RB32]	157	100.00	8.90E-88
75	YP_009148524.1	hypothetical protein [Escherichia phage HY01]	58	100.00	3.80E-28
76	YP_009148525.1	peptidase inhibitor domain-containing protein [Escherichia phage HY01]	148	98.65	1.80E-82
77	NP_049695.1	hypothetical protein [Enterobacteria phage T4]	51	100.00	1.00E-24

78	YP_009290349.1	hypothetical protein [Escherichia phage vB_EcoM-UFV13]	106	97.17	4.60E-56
79	YP_009110902.1	gp95 [Shigella phage pSs-1]	56	98.21	5.50E-24
80	APC45000.1	hypothetical protein [Shigella phage SH7]	70	100.00	8.70E-35
81	YP_007004467.1	thioredoxin [Escherichia phage ime09]	87	98.85	4.70E-46
82	YP_009102559.1	hypothetical protein [Escherichia phage ECML-134]	105	96.19	1.10E-54
83	YP_009281425.1	hypothetical protein [Escherichia phage UFV-AREG1]	309	97.41	7.40E-171
84	YP_009281426.1	hypothetical protein [Escherichia phage UFV-AREG1]	333	97.00	4.40E-185
85	YP_007004472.1	hypothetical [Escherichia phage ime09]	342	95.91	2.20E-184
86	YP_009281428.1	hypothetical protein [Escherichia phage UFV-AREG1]	296	97.64	2.40E-163
87	YP_009110911.1	hypothetical protein [Shigella phage pSs-1]	133	98.50	1.70E-68
88	YP_009110912.1	hypothetical protein [Shigella phage pSs-1]	175	99.43	1.90E-91
89	YP_007004476.1	hypothetical protein [Escherichia phage ime09]	100	100.00	1.00E-52
90	YP_006986644.1	hypothetical protein [Escherichia phage vB_EcoM_ACG-C40]	322	99.69	5.70E-182
91	YP_009148540.1	hypothetical protein [Escherichia phage HY01]	161	99.38	2.10E-84
92	YP_009281434.1	hypothetical protein [Escherichia phage UFV-AREG1]	336	98.21	2.60E-193
93	YP_007004479.1	hypothetical protein [Escherichia phage ime09]	153	98.69	2.40E-82
94	YP_009281436.1	hypothetical protein [Escherichia phage UFV-AREG1]	173	98.84	3.50E-93
95	YP_009110919.1	hypothetical protein [Shigella phage pSs-1]	177	97.74	4.10E-97
96	YP_007004482.1	hypothetical protein [Escherichia phage ime09]	57	98.25	7.30E-24
97	YP_002854061.1	hypothetical protein [Enterobacteria phage RB51]	64	100.00	1.30E-29
98	YP_004415000.1	hypothetical protein [Shigella phage Shfl2]	67	98.51	8.90E-29
99	YP_006986658.1	hypothetical protein [Escherichia phage vB_EcoM_ACG-C40]	62	98.39	1.20E-27
100	YP_803046.1	hypothetical protein [Enterobacteria phage RB32]	128	99.22	8.30E-68
101	YP_009210294.1	hypothetical protein [Escherichia phage slur02]	97	98.97	9.50E-48
102	YP_009286465.1	hypothetical protein [Salmonella phage vB_SnwM_CGG4-1]	70	65.71	3.40E-15
103	YP_803049.1	thymidine kinase [Enterobacteria phage RB32]	193	99.48	5.20E-106
104	YP_803050.1	hypothetical protein [Enterobacteria phage RB32]	62	91.94	4.70E-24
105	YP_009289180.1	hypothetical protein [Shigella phage SHSML-52-1]	63	95.08	1.00E-26
106	YP_004415009.1	hypothetical protein [Shigella phage Shfl2]	70	97.14	4.90E-38
107	YP_007004492.1	phage protein [Escherichia phage ime09]	159	100.00	1.50E-90

108	YP_009281451.1	valyl-tRNA synthetase modifier [Escherichia phage UFV-AREG1]	113	99.12	9.00E-58
109	YP_009110937.1	hypothetical protein [Shigella phage pSs-1]	181	100.00	1.50E-99
110	YP_803058.1	site-specific RNase [Enterobacteria phage RB32]	153	100.00	3.80E-83
111	YP_004415014.1	hypothetical protein [Shigella phage Shfl2]	92	97.83	1.80E-43
112	APC45026.1	hypothetical protein [Shigella phage SH7]	88	100.00	1.00E-45
113	YP_004415016.1	hypothetical protein [Shigella phage Shfl2]	73	98.63	2.80E-36
114	YP_002854079.1	hypothetical protein [Enterobacteria phage RB51]	120	100.00	4.40E-63
115	YP_009167935.1	hypothetical protein [Escherichia phage AR1]	109	99.08	2.30E-58
116	YP_009148565.1	endoribonuclease RegB domain-containing protein [Escherichia phage HY01]	179	98.88	4.20E-102
117	YP_009056703.1	internal head protein [Escherichia phage vB_EcoM_PhAPEC2]	192	96.88	1.30E-104
118	YP_009290390.1	hypothetical protein [Escherichia phage vB_EcoM-UFV13]	105	98.10	9.60E-54
119	YP_009102332.1	lysozyme murein hydrolase [Enterobacteria phage RB27]	164	99.39	1.70E-89
120	YP_009167939.1	nudix hydrolase [Escherichia phage AR1]	146	98.63	5.20E-82
121	YP_009148570.1	transmembrane region domain-containing protein [Escherichia phage HY01]	162	98.77	2.30E-91
122	YP_006986681.1	hypothetical protein [Escherichia phage vB_EcoM_ACG-C40]	124	98.39	2.10E-60
123	YP_007004509.1	predicted membrane protein [Escherichia phage ime09]	130	99.23	1.90E-64
124	YP_009149375.1	hypothetical protein [Yersinia phage phiD1]	200	98.50	1.20E-110
125	NP_049742.1	hypothetical protein [Enterobacteria phage T4]	197	99.49	6.50E-96
126	YP_002854470.1	hypothetical protein [Enterobacteria phage RB14]	58	96.55	3.90E-25
127	YP_007004513.1	phage protein [Escherichia phage ime09]	87	100.00	1.60E-46
128	YP_007004514.1	hypothetical protein [Escherichia phage ime09]	187	97.33	5.80E-94
129	YP_009277509.1	homing endonuclease [Shigella phage SHFML-11]	225	97.29	7.10E-123
130	ANH49727.1	hypothetical protein [Escherichia phage PE37]	95	98.95	4.40E-50
131	YP_009180642.1	hypothetical protein [Escherichia phage slur14]	60	98.33	5.00E-23
132	YP_009202874.1	hypothetical protein [Escherichia phage QL01]	74	87.84	5.70E-29
133	YP_803081.1	hypothetical protein [Enterobacteria phage RB32]	102	94.12	1.90E-46
134	YP_009281548.1	hypothetical protein [Escherichia phage UFV-AREG1]	90	94.44	3.60E-41
135	NP_049749.1	IpI internal head protein [Enterobacteria phage T4]	95	97.89	8.10E-44

136	NP_049750.1	hypothetical protein [Enterobacteria phage T4]	151	100.00	9.80E-84
137	YP_002854101.1	chaperone long and short tail fiber assembly [Enterobacteria phage RB51]	80	98.75	1.00E-31
138	YP_009153743.1	dNMP kinase [Yersinia phage PST]	241	99.17	1.00E-135
139	AAA50419.1	glycoprotein [Enterobacteria phage T4]	176	100.00	1.80E-97
140	YP_007004525.1	phage DNA end protector during packaging [Escherichia phage ime09]	274	99.64	2.50E-154
141	NP_049755.1	head completion protein [Enterobacteria phage T4]	150	99.33	1.40E-85
142	YP_004415043.1	putative baseplate wedge completion [Shigella phage Shfl2]	196	99.49	9.90E-113
143	YP_007004528.1	phage baseplate hub [Escherichia phage ime09]	575	99.83	0.00E+00
144	YP_007004529.1	phage protein [Escherichia phage ime09]	164	99.39	1.10E-88
145	YP_009098536.1	hypothetical protein [Enterobacteria phage RB3]	97	98.97	1.70E-49
146	YP_009102357.1	baseplate wedge subunit [Enterobacteria phage RB27]	660	99.85	0.00E+00
147	YP_009210340.1	putative baseplate wedge initiator [Shigella phage Shfl2]	1032	98.84	0.00E+00
148	YP_002854490.1	base plate wedge [Enterobacteria phage RB14]	334	99.40	9.40E-204
149	APU02787.1	baseplate wedge tail fiber connector [Escherichia phage YUEEL01]	288	99.65	9.70E-157
150	YP_009284017.1	putative baseplate wedge completion tail pin [Escherichia phage HY03]	601	99.50	0.00E+00
151	YP_803100.1	base plate wedge completion tail pin [Enterobacteria phage RB32]	219	98.63	9.40E-120
152	YP_002854116.1	short tail fibers [Enterobacteria phage RB51]	518	97.88	8.00E-295
153	YP_009288525.1	fibritin (wac) protein [Shigella phage SHBML-50-1]	485	94.63	9.90E-255
154	YP_009102365.1	neck protein [Enterobacteria phage RB27]	309	100.00	4.50E-184
155	YP_803104.1	head completion [Enterobacteria phage RB32]	256	99.61	2.50E-148
156	YP_009284023.1	tail sheath stabilizer and completion protein [Escherichia phage HY03]	272	100.00	8.30E-158
157	YP_004415058.1	putative terminase DNA packaging enzyme [Shigella phage Shfl2]	164	99.39	1.60E-87
158	YP_004415059.1	putative terminase subunit nuclease and ATPase [Shigella phage Shfl2]	610	100.00	0.00E+00
159	YP_009288531.1	tail sheath monomer [Shigella phage SHBML-50-1]	659	99.54	0.00E+00
160	YP_803109.1	tail tube monomer [Enterobacteria phage RB32]	163	100.00	8.00E-92
161	YP_803110.1	portal vertex of the head [Enterobacteria phage RB32]	524	100.00	8.60E-305
162	YP_803111.1	prohead core [Enterobacteria phage RB32]	80	100.00	1.50E-27
163	YP_009281505.1	prohead assembly protein [Escherichia phage UFV-AREG1]	141	98.58	2.90E-66

164	NP_049785.1	prohead core scaffold protein and protease [Enterobacteria phage T4]	212	99.53	2.40E-120
165	YP_803114.1	prohead core scaffold protein [Enterobacteria phage RB32]	269	99.63	2.80E-134
166	YP_009149417.1	phage major capsid protein [Yersinia phage phiD1]	521	99.23	3.30E-296
167	YP_001595302.1	hypothetical protein [Escherichia phage JS98]	88	98.86	8.90E-45
168	YP_009148616.1	head vertex protein [Escherichia phage HY01]	427	100.00	3.90E-239
169	YP_004415069.1	putative RNA ligase [Shigella phage Shfl2]	334	100.00	7.40E-193
170	YP_803118.1	hypothetical protein [Enterobacteria phage RB32]	92	100.00	1.50E-47
171	YP_009098562.1	hypothetical protein [Enterobacteria phage RB3]	61	100.00	8.40E-26
172	YP_009148620.1	putative head outer capsid protein, Hoc [Escherichia phage HY01]	283	92.59	6.50E-89
173	YP_009281514.1	minor capsid protein inhibitor of protease [Escherichia phage UFV-AREG1]	226	98.67	9.40E-123
174	YP_004415074.1	putative homing endonuclease [Shigella phage Shfl2]	205	98.05	3.20E-114
175	YP_004415075.1	putative RNA-DNA and DNA-DNA helicase ATPase [Shigella phage Shfl2]	502	99.80	2.90E-297
176	NP_049796.1	UvsW RNA-DNA and DNA-DNA helicase, ATPase [Enterobacteria phage T4]	76	100.00	1.40E-33
177	NP_049797.1	hypothetical protein [Enterobacteria phage T4]	55	100.00	4.90E-25
178	YP_009148625.1	hypothetical protein [Escherichia phage HY01]	74	100.00	2.00E-37
179	YP_004415079.1	putative ssDNA binding recombination repair protein [Shigella phage Shfl2]	137	99.27	6.60E-71
180	YP_004415080.1	putative baseplate wedge subunit [Shigella phage Shfl2]	132	100.00	2.00E-69
181	YP_002854143.1	base plate hub subunit [Enterobacteria phage RB51]	208	99.04	1.70E-118
182	YP_009102662.1	baseplate protein [Escherichia phage ECML-134]	249	99.60	7.60E-142
183	YP_002854524.1	base plate hub subunit [Enterobacteria phage RB14]	391	99.49	3.50E-226
184	YP_009102393.1	baseplate hub distal subunit [Enterobacteria phage RB27]	177	98.87	1.10E-97
185	YP_009148632.1	putative baseplate hub subunit tail length determinator [Escherichia phage HY01]	590	99.49	0.00E+00
186	YP_009197263.1	Tail-tube assembly protein [Escherichia phage slur07]	364	99.45	1.70E-198
187	YP_004415087.1	putative baseplate-tail tube initiator [Shigella phage Shfl2]	321	100.00	8.50E-178
188	NP_049808.1	hypothetical protein [Enterobacteria phage T4]	96	98.96	3.90E-46
189	YP_004415089.1	hypothetical protein [Shigella phage Shfl2]	685	98.39	0.00E+00

190	YP_007004573.1	NAD--protein ADP-ribosyltransferase [Escherichia phage ime09]	697	99.28	0.00E+00
191	YP_009149441.1	hypothetical protein [Yersinia phage phiD1]	62	98.39	5.30E-28
192	YP_009284262.1	putative base plate-tail tube initiator [Escherichia phage HY03]	487	98.97	1.40E-280
193	YP_004415093.1	hypothetical protein [Shigella phage Shfl2]	89	98.88	3.30E-47
194	YP_009030809.1	hypothetical protein [Escherichia phage vB_EcoM_112]	279	98.21	2.00E-167
195	YP_803142.1	hypothetical protein [Enterobacteria phage RB32]	152	97.37	2.70E-81
196	YP_004415096.1	hypothetical protein [Shigella phage Shfl2]	68	100.00	6.40E-35
197	YP_009148644.1	hypothetical protein [Escherichia phage HY01]	65	100.00	1.40E-31
198	YP_009111022.1	hypothetical protein [Shigella phage pSs-1]	95	97.89	5.40E-48
199	YP_002854540.1	hypothetical protein [Enterobacteria phage RB14]	122	99.18	2.30E-67
200	YP_009102679.1	hypothetical protein [Escherichia phage ECML-134]	110	98.18	1.10E-57
201	P17310.1	hypothetical protein [Enterobacteria phage T4]	72	100.00	1.70E-30
202	YP_009281541.1	protein rIII [Escherichia phage UFV-AREG1]	82	100.00	2.60E-38
203	YP_002854544.1	head assembly co-chaperonin for GroEL [Enterobacteria phage RB14]	111	98.20	4.00E-58
204	YP_004415104.1	hypothetical protein [Shigella phage Shfl2]	102	100.00	4.80E-50
205	YP_009102414.1	tail fiber protein [Enterobacteria phage RB27]	78	97.44	7.10E-38
206	YP_004415106.1	putative deoxycytidylate deaminase [Shigella phage Shfl2]	193	98.45	9.20E-111
207	YP_009281546.1	hypothetical protein [Escherichia phage UFV-AREG1]	112	100.00	3.70E-56
208	YP_004415108.1	hypothetical protein [Shigella phage Shfl2]	78	98.72	6.70E-36
209	YP_803157.1	hypothetical protein [Enterobacteria phage RB32]	91	100.00	3.00E-43
210	YP_009290482.1	hypothetical protein [Escherichia phage vB_EcoM-UFV13]	66	100.00	4.50E-33
211	YP_007004594.1	hypothetical protein [Escherichia phage ime09]	65	98.46	4.40E-33
212	YP_009148660.1	putative polynucleotide 5'-kinase and 3'-phosphatase [Escherichia phage HY01]	301	84.77	2.80E-151
213	YP_002854555.1	hypothetical protein [Enterobacteria phage RB14]	100	98.00	5.70E-48
214	YP_803160.1	hypothetical protein [Enterobacteria phage RB32]	76	100.00	2.60E-37
215	YP_009210142.1	hypothetical protein [Escherichia phage slur02]	99	98.99	1.30E-52
216	YP_006986779.1	hypothetical protein [Escherichia phage vB_EcoM_ACG-C40]	117	98.29	3.30E-55
217	YP_009098607.1	inhibitor of host transcription [Enterobacteria phage RB3]	167	98.20	1.10E-88

218	YP_009149469.1	hypothetical protein [Yersinia phage phiD1]	374	98.66	1.40E-216
219	APU02579.1	endonuclease II [Escherichia phage YUEEL01]	136	99.26	2.90E-71
220	YP_009148668.1	putative aerobic NDP reductase NrdB small subunit [Escherichia phage HY01]	392	98.72	7.80E-226
221	YP_009277602.1	ribonucleotide reductase of class Ia alpha subunit [Shigella phage SHFML-11]	754	99.60	0.00E+00
222	YP_009180729.1	hypothetical protein [Escherichia phage slur14]	95	98.95	2.10E-52
223	YP_009102703.1	hypothetical protein [Escherichia phage ECML-134]	87	96.55	7.40E-44
224	AAC12816.1	thymidylate synthase [Enterobacteria phage T4]	286	97.20	1.60E-167
225	YP_009153834.1	hypothetical protein [Yersinia phage PST]	120	96.61	1.30E-62
226	APU02587.1	dihydrofolate reductase [Escherichia phage YUEEL01]	193	96.37	4.40E-105
227	APU02588.1	hypothetical protein [Escherichia phage YUEEL01]	81	100.00	4.20E-41
228	NP_049851.1	hypothetical protein [Enterobacteria phage T4]	80	97.50	4.10E-41
229	APU02591.1	hypothetical protein [Escherichia phage YUEEL01]	121	95.04	9.00E-64
230	AAA74660.1	frd3 [Enterobacteria phage Pol]	75	97.33	4.90E-36
231	APU02593.1	single-stranded DNA binding protein [Escherichia phage YUEEL01]	302	99.67	8.80E-169
232	YP_007004615.1	phage DNA helicase loader protein [Escherichia phage ime09]	217	99.54	3.50E-119
233	YP_803179.1	late promoter transcription factor [Enterobacteria phage RB32]	112	99.11	2.10E-54
234	YP_009281578.1	double-stranded DNA binding protein [Escherichia phage UFV-AREG1]	89	98.88	8.40E-43
235	NP_049859.1	RNaseH ribonuclease [Enterobacteria phage T4]	305	100.00	3.70E-175
236	YP_007004620.1	phage long tail fiber proximal subunit [Escherichia phage ime09]	1289	96.51	0.00E+00
237	YP_009281581.1	hinge connector of long tail fiber proximal connector [Escherichia phage UFV-AREG1]	372	96.51	2.30E-203
238	YP_009180746.1	Phage T4 tail fiber [Escherichia phage slur14]	219	92.73	2.70E-111
239	AJC64544.1	gp37 [Enterobacteria phage T4]	1013	78.19	0.00E+00
240	CAA38976.1	receptor-recognizing protein 38 [Enterobacteria phage T4]	183	96.72	5.50E-105
241	YP_009281585.1	holin [Escherichia phage UFV-AREG1]	218	99.08	8.50E-121
242	YP_009290514.1	hypothetical protein [Escherichia phage vB_EcoM-UFV13]	90	98.89	3.80E-43
243	YP_009290515.1	hypothetical protein [Escherichia phage vB_EcoM-UFV13]	50	98.00	8.10E-19
244	YP_009284205.1	putative baseplate wedge tail fiber connector [Escherichia phage HY03]	92	98.91	4.20E-45

245	YP_009197202.1	hypothetical protein [Escherichia phage slur07]	98	97.96	7.80E-50
246	YP_009102458.1	hypothetical protein [Enterobacteria phage RB27]	153	98.04	2.60E-84
247	YP_009148696.1	hypothetical protein [Escherichia phage HY01]	109	100.00	6.00E-59
248	YP_009153861.1	activator of middle period transcription [Yersinia phage PST]	211	99.53	4.50E-111
249	YP_006986820.1	hypothetical protein [Escherichia phage vB_EcoM_ACG-C40]	49	100.00	6.30E-16
250	YP_009290524.1	DNA topoisomerase [Escherichia phage vB_EcoM-UFV13]	442	99.77	4.20E-252
251	YP_007004639.1	nuclear disruption protein [Escherichia phage ime09]	151	98.68	2.40E-82
252	YP_009278990.1	hypothetical protein [Shigella phage SHFML-26]	71	100.00	3.30E-34
253	YP_007005008.1	hypothetical protein [Escherichia phage wV7]	65	98.46	3.30E-28
254	YP_002854606.1	hypothetical protein [Enterobacteria phage RB14]	68	100.00	5.30E-29
255	YP_009281603.1	endonuclease IV [Escherichia phage UFV-AREG1]	185	98.92	4.10E-108
256	YP_009281604.1	hypothetical protein [Escherichia phage UFV-AREG1]	64	98.44	3.90E-26
257	YP_002854610.1	membrane-associated protein [Enterobacteria phage RB14]	312	99.68	2.70E-176
